# Diffusion tensor imaging for evaluating perianal fistula: Feasibility study

**DOI:** 10.1097/MD.0000000000011570

**Published:** 2018-07-20

**Authors:** Yu Wang, Chao Gu, Yongjun Huo, Weiwei Han, Jinfen Yu, Chengzong Ding, Xiuyu Zhao, Yunfang Meng, Chuanting Li

**Affiliations:** a Shandong Medical Imaging Research Institute, Shandong University; b Affiliated Hospital of Shandong University of Traditional Chinese Medicine; c Traditional Chinese Medical Hospital, Zhangqiu; d Shandong Provincial Hospital, Jinan, Shandong, China.

**Keywords:** activity, diffusion tensor imaging, magnetic resonance imaging, perianal fistula

## Abstract

To explore the feasibility of using diffusion tensor imaging (DTI) in the diagnosis of anal fistula and evaluating its activity.

Thirty-four patients with perianal fistulas were examined with DTI on a 3.0 T magnetic resonance imaging (MRI) before undergoing surgery. Based on the surgery requirement and preoperative examinations, the lesions fell into 2 groups: the positive inflammation activity (PIA) group and the negative inflammation activity (NIA) group. Each lesion was divided into 3 regions of interest (ROIs) (i.e., the fistula area, edema area, and distant normal-appearing area). Fractional anisotropy (FA) and apparent diffusion coefficient (ADC) values were calculated and analyzed.

There were statistically significant differences in FA and ADC values of the fistula area, edema area, and distant normal-appearing area. The FA values of the fistula area, edema area, and distant normal-appearing area in PIA were 0.134 ± 0.046, 0.225 ± 0.060, 0.343 ± 0.070, respectively. The ADC values (×10^−3^ mm^2^/s) of the fistula area, edema area, and distant normal-appearing area in PIA were 0.979 ± 0.441, 1.542 ± 0.274, 1.864 ± 0.336, respectively. The FA values of the fistula area, edema area, and distant normal-appearing area in NIA were 0.183 ± 0.057, 0.286 ± 0.059, 0.382 ± 0.084, respectively. The ADC values (×10^−3^ mm^2^/s) of the fistula area, edema area, and distant normal-appearing area in NIA were 1.393 ± 0.256, 1.518 ± 0.274, 1.703 ± 0.432, respectively. Regarding the activity, the FA and ADC values of the PIA group were lower than those of the NIA group in the fistula area, and the differences were statistically significant (*P* = .009, .004). The FA values of the edema area in the PIA group were lower than those in the NIA group, and the difference was statistically significant. The ADC values of the edema area, and both the FA and ADC values of the distant normal-appearing area all exhibited no statistically significant differences between the 2 groups.

DTI parameters may reflect microstructures of perianal fiatulas via quantitative information. FA and ADC values were instrumental in evaluating the activity of perianal fistulas.

## 1 Introduction

Perianal fistulas are a common disease in department of anorectal surgery. The incidence is actually higher than that recorded in the literature.^[1]^ Accurate assessment of fistular activity was an important factor influencing therapeutic strategy, such as in the selection of the number of required drugs, the choice of operation time, and the type of surgical approach.^[2]^

Magnetic resonance imaging (MRI) is highly accurate for the detection of both the primary tracts of fistulas and the formation of abscesses in patients living with perianal diseases.^[3]^ Apart from its usefulness in the anatomic investigation of perianal fistulas, MRI can be used to evaluate perianal fistular activity, a significant factor in therapeutic strategy determination.^[4,5]^

Quite a few studies have discussed the role of diffusion-weighted imaging (DWI) sequences in the diagnosis and differential diagnosis of anal fistulas and perianal abscesses,^[6–9]^ and many studies^[4,10]^ have also evaluated the perianal fistular activity. However, no study has described the application of diffusion tensor imaging (DTI) to this issue. Our study retrospectively explored the pathological features of fistula patients, and analyzed the value of DTI sequence in the evaluation of perianal fistular activity.

## 2 Materials and methods

### 2.1 Study population

From July 2016 to June 2017, 34 patients with perianal fistula, from the Affiliated Hospital of Shandong University of Traditional Chinese Medicine, were examined in the study (29 men, 5 women; average age, ∼39.6 years; range, 18–60 years old). The clinical manifestations were perianal swelling and heat pain, lasting from 10 days, at the shortest, to 4 months, at the longest. All patients underwent routine MRI sequences and DTI sequences in a 3.0T MR scanner (Intera, Philips Medical Systems, Philips Achieva TX, Best, Amsterdam, the Netherlands) before surgery. Every patient was informed of the condition before the examination, and informed consent was obtained, both of which were approved by the ethics committee. The need for fistular surgery was based on the following symptoms: discharge of pus, severe heat pain, reddish and swollen skin, and increased serum C-reactive protein (CRP) levels (>2 mg/L). Clinical examinations confirmed the opened external orifice of the fistula, and palpable subcutaneous fibrous cords. Surgery was performed under general anesthesia. If pus was confirmed during surgery, the fistulas were assumed to be active and were classified in the positive inflammation activity (PIA) group. Nevertheless, if pus was absent during surgery, or if surgery seemed unnecessary, the fistulas were classified in the negative inflammation activity (NIA) group. Based on surgical findings, the lesions fell into 1 of the 2 groups, namely, the PIA group or the NIA group.

### 2.2 Techniques and methods

All subjects were examined by 3.0T MRI. A 16-channel surface coil was used to cover the entire pelvic area, with subjects in a supine position with the center of pubic bone the legs parallel, lightly flexed. Subjects were required to have had an empty stomach for 6 hours and empty their bladders before the examinations.

The MRI protocol contained 4 sequences: turbo spin echo (TSE) T1-weighted imaging (T1WI): axial; TR/TE, 600/10 ms; field of view (FOV), 200 × 200 mm^2^; matrix, 400 × 400; slice thickness, 5 mm; and number of slices, 20. TSE T2-weighted imaging (T2WI): axial; TR/TE, 1560/80 ms; FOV, 200 × 200 mm^2^; matrix, 400 × 400; slice thickness, 5 mm; and number of slices, 20. Fat-suppression T2-weighted imaging (FS-T2WI): sagittal; 152 × 179 mm^2^; matrix, 236 × 147 mm^2^; slice thickness, 5 mm; and number of slices, 20. DTI (spin echo-echo-planar imaging, SE-EPI): axial; TR/TE, 3250/48 ms; FOV, 200 × 200 mm^2^; matrix, 80 × 80 with a 112 × 112 reconstructed matrix; slice thickness, 5 mm; number of slices, 20; 32 diffusion-weighed directions; NSA, 1; *b* = 0.400 s/mm^2^; spectral adiabatic inversion recovery (SPAIR) for fat suppression; and imaging time, 5 minutes 47 seconds.

### 2.3 Image post-processing and measurements

The original DTI data were transferred to an independent workstation and were processed by software FA and ADC maps. Two experienced radiologists evaluated the quality of image, discussed to settle any disagreement, and measured the FA and ADC values of the imaged regions of interest (ROIs). ROIs were delineated manually on DTI (*b* = 0) images on the largest suspected area, and overlaid on FA and ADC maps, and the average value was obtained by measuring 3 times. The minimum size used for measurements was 10 mm^2^. In addition, the lesions were divided into 3 ROIs (i.e., the fistula area, edema area, and distant normal-appearing area). The puborectalis was chosen to represent normal-appearing area in our study. Three ROIs were placed in each lesion: the first ROI was placed in the center of lesion and was marked as the fistula area; the second ROI was placed on the edge of lesion and was marked as the edema area; and the third ROI was placed on the unaffected puborectalis muscle and was marked as distant, normal-appearing area (ab. normal area).

### 2.4 Statistical analysis

All statistical analyses were conducted with the Statistical Package for Medical Statistics (Medcalc 15.8, Ostend, Belgium; https://www.medcalc.org). All the FA, ADC values are presented with mean ± standard deviation (*x* ± *s*). The FA and ADC values of each area were calculated and analyzed with single-factor analysis of variance (ANOVA). The DTI values of each group were statistically calculated with independent samples *t* tests. The cut-off values for the ADC and FA measurements in evaluating the activity of the lesions were obtained by receiver operating characteristic (ROC) curve analysis, as were the sensitivity and specificity. In all tests, *P* < .05 represented statistically significant differences.

## 3 Results

### 3.1 Classification of fistulas and the MR signal

The main fistula was chosen for measurement in each lesion, and was classified according to the classification system of Parks et al (Table 1).^[11]^ Most of the fistula areas and edema areas exhibited hypointensity on T1WI (26/34, 34/34, respectively); and hyperintensity on T2WI, FS-T2WI, and DTI (29/34, 29/34, 34/34, respectively). The intensity of edema area was significantly lower than that of the fistula area. The fistula area showed hypointensity on the FA and ADC maps (34/34, 30/34, respectively), and the edema area showed isointensity (Table 2).

**Table 1:**
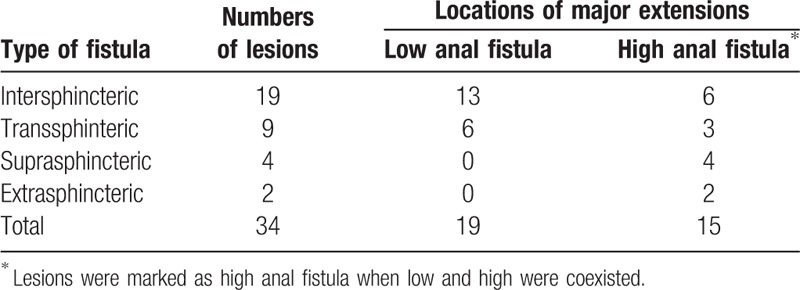
Classification of fistulas according to the Parks system.

**Table 2:**
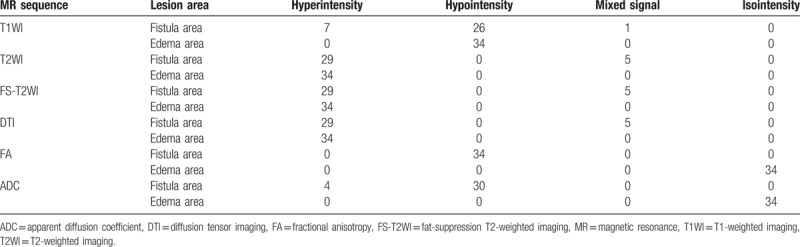
Magnetic resonance signal.

### 3.2 The DTI parameter values of each lesion area

**Figure 1:**
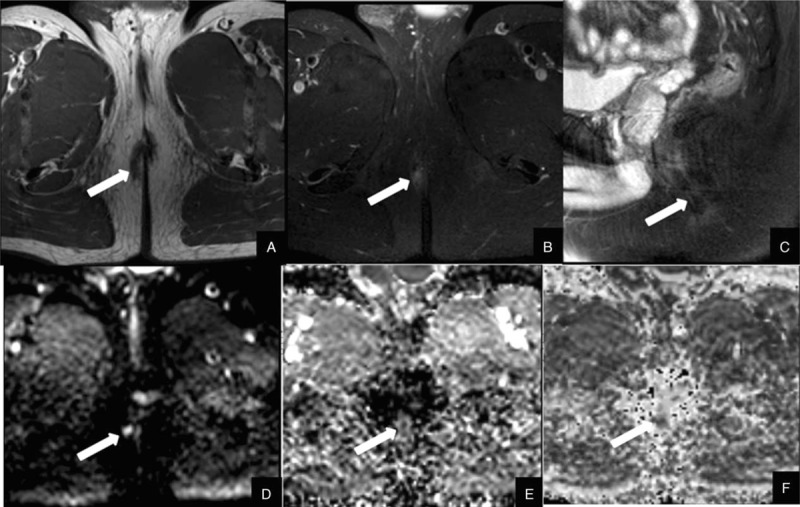
A 39-year-old man with subcutaneous anal fistula of negative inflammation activity. (A) Axial T2-weighted image shows a perianal fistula (arrow) that exhibits equal and slightly high signal intensity. (B and C) Axial and sagittal fat-suppression T2-weighted images show a perianal fistula (arrow) that exhibits high signal intensity. (D) Axial diffusion tensor imaging reveals high signal intensity at the fistula (arrow). (E) Axial apparent diffusion coefficient (ADC) map shows a value of 1.149 × 10^−3^ mm^2^/s for the fistula. (F) Axial fractional anisotropy (FA) map shows a value of 0.13.

**Figure 2:**
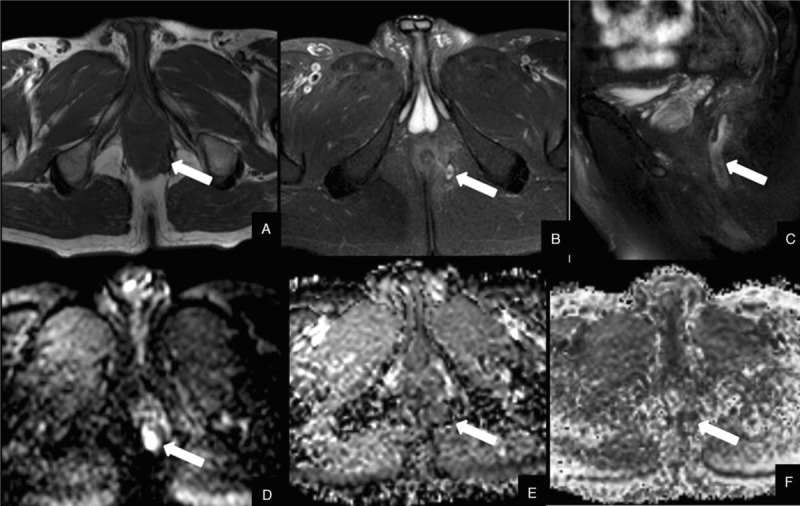
A 34-year-old man with transsphinteric anal fistula of positive inflammation activity with abscess. (A) Axial T2-weighted image shows a perianal fistula (arrow) that exhibits slightly high signal intensity. (B and C) Axial and sagittal fat-suppression T2-weighted images show a perianal fistula (arrow) that exhibits high signal intensity. (D) Axial diffusion tensor imaging reveals high signal intensity at the fistula (arrow). (E) Axial apparent diffusion coefficient (ADC) map shows a value of 0.377 × 10^−3^ mm^2^/s for the fistula. (F) Axial fractional anisotropy (FA) map shows a value of 0.073.

There were statistical significant differences between the FA and ADC values between 3 regions of interest in both groups. The FA values of the fistula areas, edema area, and distant normal-appearing area in PIA group were 0.134 ± 0.046, 0.225 ± 0.060, 0.343 ± 0.070, respectively. The ADC values (×10^−3^ mm^2^/s) of the fistula areas, edema area, and distant normal-appearing area in PIA were 0.979 ± 0.441, 1.542 ± 0.274, 1.864 ± 0.336, respectively. The FA values of the fistula areas, edema area, and distant normal-appearing area in NIA were 0.183 ± 0.057, 0.286 ± 0.059, 0.382 ± 0.084, respectively. The ADC values (×10^−3^ mm^2^/s) of the fistula areas, edema area, and distant normal-appearing area in NIA were 1.393 ± 0.256, 1.518 ± 0.274, 1.703 ± 0.432, respectively. In regard to activity, the FA and ADC values of the PIA group were lower than those of the NIA group in the fistula area, and the differences were statistically significant (*P* = .009, .004, Figs. 1 and 2). The FA values of edema areas of the PIA were lower than those of the NIA, and the differences were statistically significant. The ADC values in the edema area and both the FA and ADC values in the distant normal-appearing area exhibited no significant differences between the 2 groups (Table 3).

**Table 3:**
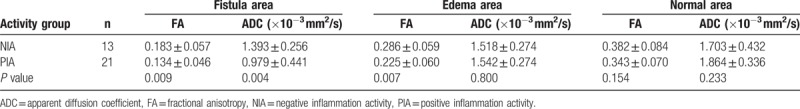
The diffusion tensor imaging parameter values of each lesion area.

### 3.3 The cut-off, sensitivity, and specificity of the FA and ADC values in the differential diagnosis of perianal fistular activity

**Figure 3:**
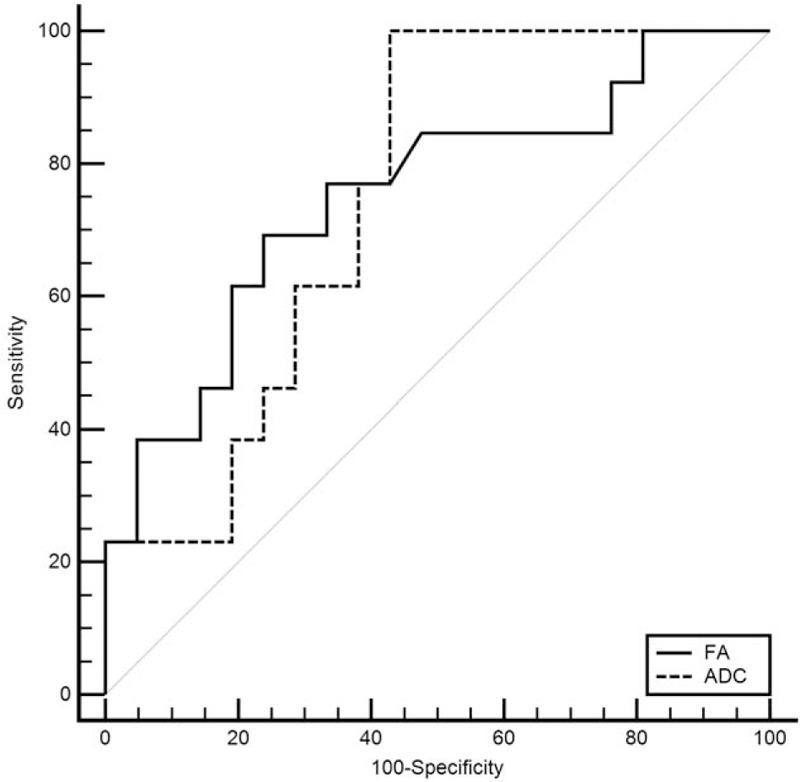
The receiver-operating characteristic (ROC) analysis of 2 parameters for the diagnosis of abscess area in perianal fistulas, respectively, in the PIA and NIA group: the area under the ROC curve (AUC) below the black dotted line is <0.5. The AUC of ADC is bigger than that of FA, the ADC value is more sensitive than FA, however, FA value is more specific.

**Figure 4:**
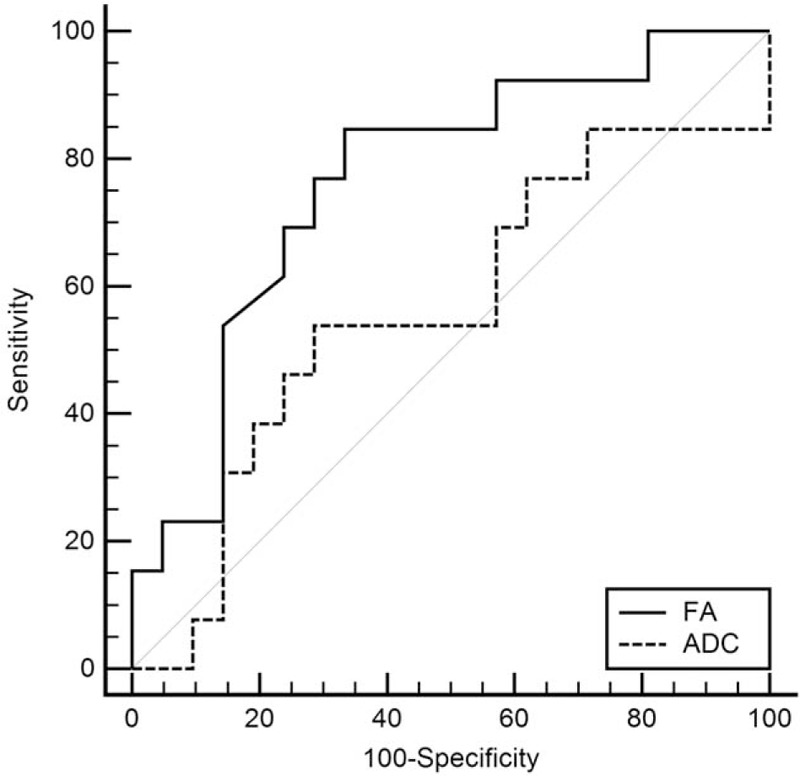
The receiver-operating characteristic (ROC) analysis of 2 parameters for the diagnosis of edema area in perianal fistulas, respectively, in the PIA and NIA group: the area under the ROC curve (AUC) below the black dotted line is <0.5. The AUC of FA is bigger than that of ADC, however, the ADC value has no statistical significance. The FA value is more sensitive.

To obtain the cut-off values of the 2 parameters for the differential diagnosis of the activity of perianal fistulas, ROC analyses were carried out, and the sensitivity and specificity were calculated at the same time. The cut-off points of ADC, FA values in PIA were 1.069, 0.15 in the fistula area and 1.444, 0.25 in the edema area. The sensitivity and specificity for distinguishing PIA and NIA were 100.0% and 57.14% for ADC, 69.23% and 76.19% for FA (Table 4, Figs. 3 and 4).

**Table 4:**
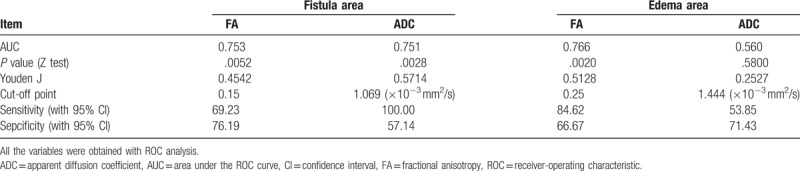
Variables of receiver-operating characteristic analysis for the 2 parameters analyzed for the differential diagnosis of negative inflammation activity and positive inflammation activity.

## 4 Discussion

Perianal fistula is a common surgical disease, and there is a high recurrence rate because the potential infections are often ignored during the operation. Preoperative imaging particularly MRI findings have been reported to play important roles in detection of those unidentified infections, thus are essential for diminishing the chance of recurrence.^[12]^ Our results confirmed the important role of MRI in detection of the infection; furthermore, our results indicated that in addition to ADC, DTI with FA and ADC may play a role in determining the range of lesions and the activity of perianal fistulas.

DTI is a type of functional imaging based on DWI, which was originally applied to the central nervous system,^[13,14]^ and is mainly used to display the pathology and physiology of white matter tracts. At present, DTI is used in organs in an increasing number of cases apart from the central nervous system, such as cases of renal tubules^[15,16]^ and muscle tissue,^[17]^ in the determination of the feasibility of normal woman pelvic structure^[18]^ and in studies of DTI of pelvic floor dysfunction.^[19]^ In our study, we explored the feasibility of using DTI in the diagnosis of perianal fistulas and the value of DTI in the evaluation of fistular activity.

DTI can be used to express the degree and direction of the random Brownian motion of water molecules in the body, and the commonly used indices are the FA and ADC values. The FA value reflects the direction of movement of water molecules and ranges from 0 to 1. A value of 0 indicates that the water diffusion is isotropic and that the diffusion amplitudes are the same in all directions, whereas values closer to 1 indicate that the water molecules are spread more strongly in different directions. Due to the different microstructures of different tissues, the movements of the diffusion of water molecules are also different, which are manifested by the anisotropic diffusion, and it is especially evident in organizational structures. The perianal muscles are symmetrical on both sides of the structure, while the sphincter complex of the anal canal is mainly circular or U-shaped. When anal fistulas destroy the perianal muscle structure, the diffusion direction of water molecules also changes, becoming disorderly and irregular and exhibiting decreases in FA values. The decreased FA indicates that the local structure filled with inflammatory cells is disorganized and disorderly. Due to the oppression of the fistula area, the FA value of the peripheral edema area also decreased, but no organizational necrosis had yet occurred, so the FA value was relatively high. The measurement of DTI tensor values could be adopted as a reference for surgeons to determine the scope of the operation. Values between ∼0.065 to 0.265 and ∼0.377 to 1.851 were roughly the ranges we measured for the FA and ADC values, respectively, of the fistula area; the ranges of the FA and ADC values of the edema area were ∼0.102 to 0.386 and ∼0.927 to 1.970, respectively. The ranges had some overlap since the necrotic lesions in the pathological process could not be completely separated. Zijta et al^[18]^ reported the DTI tensor values of normal pelvic floor muscles. However, the FA values of perianal fistulas had not yet been reported. The ADC value is an indicator of irregular Brownian motion of water molecules in tissue. The larger the ADC value, the stronger the ability to exercise, and the greater the extent of the irregular movement within the unit time. Pus is a viscous liquid containing a large number of inflammatory cells, bacteria, necrotic tissue, and protein secretions. A high viscosity and a large number of inflammatory cells limit the diffusion of water molecules and induced decreases in the ADC value. We measured the ADC (×10^−3^ mm^2^/s) value of the fistula area in the PIA group and found that it was 0.979 ± 0.441, which was close to the value reported in the literature (0.908 ± 0.171).^[4]^ Additionally, the ADC value of the fistula area in the NIA group was 1.393 ± 0.256, which was slightly higher than the reported value (1.124 ± 0.244).^[4]^

As for the activity of the disease, variable concentrations of inflammatory cells and bacteria appear in different pathogenic organisms. The host immune response and the age of the fistula might influence the viscosity of the pus. Fistular composition changes the relative stability of water molecule diffusion to a certain extent. The NIA group demonstrated a slightly higher FA value, which reflected viable inflammatory cells organized and oriented in the fistula area. Reasons such as the viscosity of micromolecules and reduced cellular gaps could lead to the restricted diffusion of water molecules and thus to the reduction of ADC values. As a result, the NIA group exhibited a reduced ADC value. These findings were all consistent with our results. Nevertheless, only the data regarding the fistula area was statistically significant, and there were no statistically significant differences observed in the edema areas or the normal areas. This result may have been related to the selected ROI, and further work should be done to improve the research.

We admit that there were many deficiencies in our research. First, we had a relatively small sample size. Second, we recruited more male patients than female patients. The sex ratio imbalance may have affected the results of the study, which also reflected that the incidence of anal fistulas is higher in men than in women. Third, as for the artifacts on the pelvic floor, in order to guarantee feasibility, we first chose the low value of 400 (s/mm^2^) as the *b*-value, but in the future, it may be appropriate to acquire more *b*-values to obtain more accurate values. Fourth, we chose the puborectalis muscle as the reference standard because this muscle is easy to identify. In fact, the muscle most closely related to anal fistulas is the anal sphincter; however, the anal sphincter often undergoes pathological changes. Fifth, the applied reference standard, which was based on the need for surgery (which was determined clinically) and the surgical findings, may be regarded as imperfect. In this sense, the results are only preliminary, and further investigations are needed.

## 5 Conclusions

DTI parameters may reflect microstructures of perianal fiatulas by quantitative information. FA and ADC values are important for anorectal surgeon to use as references during surgery to evaluate the activity of perianal fistulas.

## Author contributions

**Conceptualization:** Yu Wang, Chao Gu, Chuanting Li.

**Data curation:** Yu Wang, Chao Gu, Yongjun Huo, Weiwei Han, Jinfen Yu.

**Formal analysis:** Yu Wang, Chao Gu, Jinfen Yu.

**Investigation:** Yu Wang, Chao Gu, Weiwei Han, Yunfang Meng.

**Methodology:** Yu Wang, Chao Gu, Yongjun Huo, Jinfen Yu, Chengzong Ding, Yunfang Meng, Chuanting Li.

**Project administration:** Yu Wang, Chao Gu, Yongjun Huo, Weiwei Han, Jinfen Yu, Chengzong Ding, Xiuyu Zhao, Chuanting Li.

**Resources:** Yu Wang, Chao Gu, Yongjun Huo, Weiwei Han, Xiuyu Zhao, Chuanting Li.

**Software:** Chao Gu, Yongjun Huo, Weiwei Han, Xiuyu Zhao, Yunfang Meng, Chuanting Li.

**Supervision:** Chengzong Ding, Yunfang Meng, Chuanting Li.

**Writing – original draft:** Yu Wang, Chao Gu, Weiwei Han.

**Writing – review and editing:** Yu Wang, Chao Gu, Weiwei Han, Jinfen Yu, Yunfang Meng, Chuanting Li.
